# STOUT V2.0: SMILES to IUPAC name conversion using transformer models

**DOI:** 10.1186/s13321-024-00941-x

**Published:** 2024-12-27

**Authors:** Kohulan Rajan, Achim Zielesny, Christoph Steinbeck

**Affiliations:** 1 Institute for Inorganic and Analytical Chemistry, Friedrich Schiller University Jena, Lessingstr. 8, 07743 Jena, Germany; 2 Institute for Bioinformatics and Chemoinformatics, Westphalian University of Applied Sciences, August-Schmidt-Ring 10, 45665 Recklinghausen, Germany

**Keywords:** Transformers, STOUT, SMILES to IUPAC name, Chemical name translation, Deep learning

## Abstract

Naming chemical compounds systematically is a complex task governed by a set of rules established by the International Union of Pure and Applied Chemistry (IUPAC). These rules are universal and widely accepted by chemists worldwide, but their complexity makes it challenging for individuals to consistently apply them accurately. A translation method can be employed to address this challenge. Accurate translation of chemical compounds from SMILES notation into their corresponding IUPAC names is crucial, as it can significantly streamline the laborious process of naming chemical structures. Here, we present STOUT (SMILES-TO-IUPAC-name translator) V2, which addresses this challenge by introducing a transformer-based model that translates string representations of chemical structures into IUPAC names. Trained on a dataset of nearly 1 billion SMILES strings and their corresponding IUPAC names, STOUT V2 demonstrates exceptional accuracy in generating IUPAC names, even for complex chemical structures. The model's ability to capture intricate patterns and relationships within chemical structures enables it to generate precise and standardised IUPAC names. While established deterministic algorithms remain the gold standard for systematic chemical naming, our work, enabled by access to OpenEye’s Lexichem software through an academic license, demonstrates the potential of neural approaches to complement existing tools in chemical nomenclature.

**Scientific contribution **STOUT V2, built upon transformer-based models, is a significant advancement from our previous work. The web application enhances its accessibility and utility. By making the model and source code fully open and well-documented, we aim to promote unrestricted use and encourage further development.

**Graphical Abstract:**

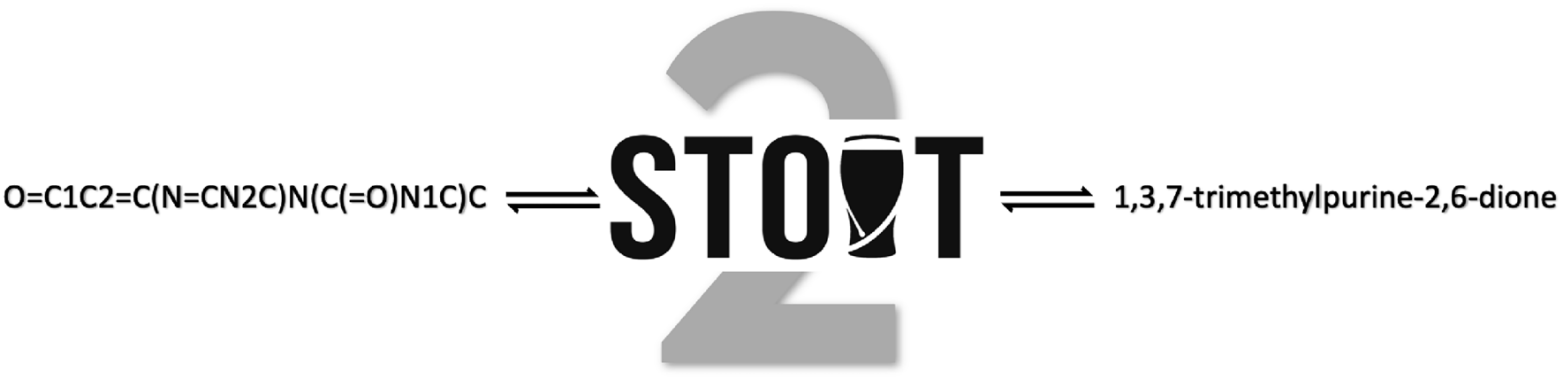

## Introduction

Chemists usually assign chemical structures a name when they are discovered or synthesised for the first time. These names can be trivial or systematic. The systematic names must follow a set of rules specified by the International Union of Pure and Applied Chemistry (IUPAC) [1–5]. These rules are comprehensive and complex, making it difficult to apply them consistently, especially for large datasets of chemical compounds or highly complex chemical structures.

Several commercial software packages are available to generate IUPAC names from chemical structures [6–9]. Chemaxon [10] and OpenEye [11] offer their rule-based software under an academic license to generate IUPAC names. This software enables chemists to create IUPAC names for specified structures automatically. Due to their deterministic algorithms, these rule-based software packages are reliable and widely used. The work presented here was only possible due to their existence (see below).

Machine learning, particularly deep neural networks, has shown promise in various domains [12, 13], including natural language processing (NLP) [14]. It has been successfully applied in tasks like language translation [15], demonstrating the ability to learn complex patterns and relationships from large datasets [16, 17].

This success has inspired researchers to explore the application of neural networks in cheminformatics, particularly in tasks like predicting chemical reactions [18], Optical Chemical Structure Recognition (OCSR) [19], drug discovery [20], generating novel molecules [21, 22], molecular design and optimising [23] and many more [24].

Recent studies have explored the use of sequence-based neural networks for translating between chemical representations, such as SMILES strings and IUPAC names [25–27]. These studies highlight the potential of current machine learning methods to tackle the challenges of IUPAC name generation. However, we have no intention of competing with commercial rule-based tools that are more reliable due to their deterministic nature.

In recent years, OPSIN (Open Parser for Systematic IUPAC Nomenclature) [28] has been developed as the only open-source rule-based system for parsing IUPAC names into SMILES strings. It uses a regular grammar approach to guide tokenisation and constructs an XML parse tree from the IUPAC name, which is then processed to reconstruct the chemical structure. In this work, we use OPSIN to retranslate the IUPAC names generated by STOUT.

In our previous work [26], we introduced STOUT (SMILES-TO-IUPAC-name translator), a deep learning model based on a sequence-to-sequence (seq2seq) architecture with an encoder and decoder using Recurrent Neural Networks (RNNs) with Gated Recurrent Units (GRUs) [26]. STOUT was trained on a dataset of 60 million molecules from PubChem [29] and corresponding IUPAC names generated with ChemAxon’s molconvert software [30]. The model achieved promising results, with an average BLEU score of approximately 90% and a Tanimoto similarity index [31] of over 0.9, indicating high accuracy in predicting IUPAC names from SMILES strings and vice versa. However, there were still areas for improvement, particularly in model architecture, tokenisation strategies, and the handling of stereochemical information.

**Fig. 1:**
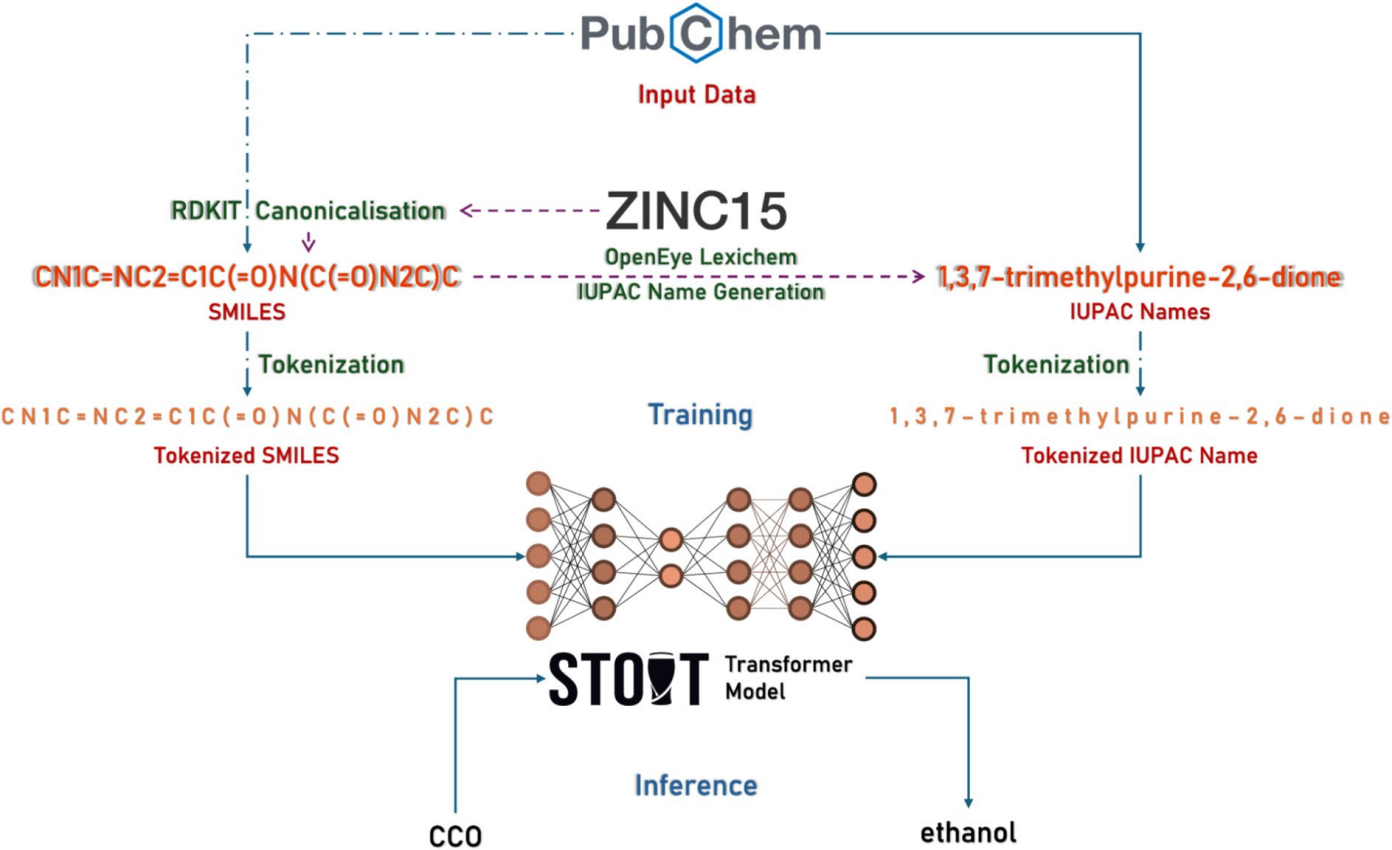
Workflow of SMILES to IUPAC name translator version 2.0

This work presents STOUT V2, a transformer-based model for SMILES to IUPAC name translation (see Fig. 1). The model was trained on a dataset of nearly 1 billion SMILES and corresponding IUPAC names, generated using OpenEye's Lexichem software. This new version achieves very high accuracy on test and benchmark datasets, demonstrating improved capability in producing longer IUPAC names with fewer errors overall. The models were trained entirely on Tensor Processing Unit (TPU) VMs, and after finalising the training, they were optimised using TensorFlow to run efficiently on CPUs and have been made available along with the accompanying code as open-source resources. To enhance accessibility for users with limited or no programming experience, a web application has been developed and is accessible at https://stout.decimer.ai. We demonstrate that neural networks can accurately perform the non-trivial task of converting SMILES to IUPAC names. However, we accomplished this with the generous access to Lexichem software that OpenEye provided us. The use of deterministic algorithms for generating IUPAC names, such as Lexichem, in production environments is always recommended due to the higher error rates associated with neural machine translation.

## Methods

In STOUT V2, the main focus was improving the model and determining the appropriate token space and the maximum length of the input and output strings during training. In addition, an appropriate string representation and the assessment of the models' performance concerning training set size were investigated.

### Datasets

Most of the approaches in this work were carried out using structures from PubChem, one of the largest databases for chemical structures. For large-scale training and analysis, the whole ZINC 15 database was used. PubChem was primarily selected because it publishes SMILES strings of chemical structures and their IUPAC names. PubChem contains IUPAC names systematically generated using the OpenEye Lexichem TK 2.7.0 software [32].

### Training dataset generation

The training datasets were downloaded from the respective data sources as SMILES. The downloaded SMILES strings were parsed through RDKit version 2023.03.1 to obtain kekulised canonical SMILES and stereochemical information. OpenEye Lexichem TK 2.8.1 was used for generating IUPAC names from SMILES strings. For SMILES preprocessing, RDKit version 2023.03.1 was consistently used across all datasets. It should be noted that STOUT is trained exclusively on standardised IUPAC names, not on synonyms. All IUPAC names downloaded from PubChem are generated by Lexichem, which ensures consistency in the training data.

#### Experiment 1: Impact of dataset size and tokenisation on model performance

This experiment investigated the impact of dataset size and tokenisation on model performance using data from the PubChem database. The SMILES of each chemical structure and its system-generated IUPAC name were directly downloaded from PubChem and used as-is, with all stereochemical information retained.

From the 110 million compounds downloaded from PubChem [29] using the chemfp [33] implementation of the MaxMin [34] algorithm, approximately 51 million were selected as a subset. From this subset, a training dataset of 50 million compounds and a testing dataset of 1 million compounds were chosen. Furthermore, from the 50 million subset, an additional 11 million compound subset was selected using the MaxMin algorithm. From this, a training set of 10 million compounds and a test set of 1 million compounds were selected. As a final step, from the 11 million compounds subset, a 1 million compound training set and a 250,000 test set were selected using the MaxMin algorithm. The MaxMin algorithm is used to select diverse training and test sets, by maximizing the minimum distance between samples to ensure diverse and representative data distribution of the same chemical space while minimizing redundancy in model development and evaluation.

**Fig. 2:**
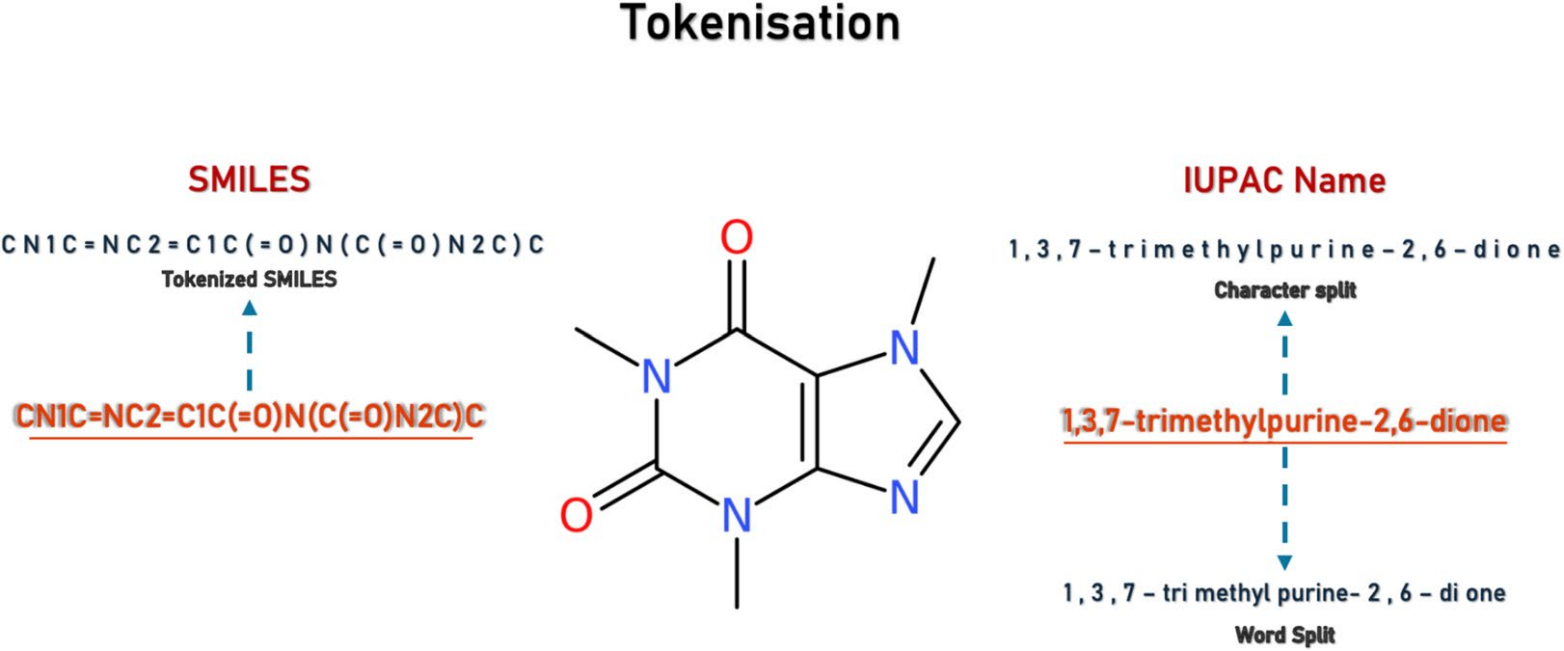
Comparison of tokenisation methods for the chemical compound caffeine. The figure illustrates SMILES representation of its respective tokenisation processes and contrasts IUPAC name character-level splitting with word-level splitting. The molecular structure image is included for reference

The tokenisation methods for SMILES followed the procedures outlined in Appendix 1 and Fig. 2. IUPAC names were tokenised both by meaningful words and character-wise, resulting in additional training datasets. Here, characterwise splitting means that each character in the IUPAC name has been separated into a token, increasing the maximum length of the tokenised IUPAC names.

The effect of increasing dataset size on the number of unique tokens and the maximum length of split strings was also analysed. Table 1 summarises the datasets used for training the models for SMILES strings to IUPAC name translations, with the IUPAC names split by characters or words. In total, 6 combinations of training datasets were generated.

**Table 1:** Summary of datasets for SMILES to IUPAC name translation with character and word splits

	IUPAC name character split					IUPAC name word split				
	Input token count	Maximum input length	Output token count	Maximum Output length	Average training time (per epoch)	Input token count	Maximum input length	Output token count	Maximum output length	Average training time (per epoch)
1 million	125	150	64	150	50 s	125	150	855	150	57 s
10 million	126	200	64	300	6 min 10 s	126	200	1199	300	7 min 8 s
50 million	132	400	66	400	55 min 43 s	132	400	1501	400	1 h 14 min 53 s

#### Experiment 2—Training with increased data and longer sequences

The final model, selected based on the results from Experiment 1, was trained on a large dataset derived from PubChem. Initially, 111,500,902 compounds and their corresponding IUPAC names were downloaded from PubChem. However, SMILES strings longer than 600 characters were removed from the training dataset along with the corresponding IUPAC names. This filtering process resulted in a refined dataset of 111,104,000 molecules.

From this refined dataset, 110 million molecules were allocated for training the model. To create a balanced train and test set, 1 million molecules were selected using the chemfp implementation of the MaxMin algorithm. This algorithm ensures a diverse selection of compounds for testing, covering a wide range of chemical spaces.

SMILES were used as input, and IUPAC names were tokenised by character. This contained 132 unique SMILES tokens with a maximum length of 600 characters, while the IUPAC name contained 68 unique tokens with a maximum length of 600 characters. Compared with the 50 million datasets, the maximum length of input and output tokens has increased by 200 characters, while the size of input and output tokens has remained relatively unchanged.

#### Experiment 3—Large-scale training and generalization analysis

The training dataset for this experiment was generated using the ZINC 15 dataset combined with PubChem molecules. The ZINC 15 dataset was downloaded as SMILES and parsed through RDKit to generate canonical SMILES with stereochemical information and kekulised. Any SMILES that did not parse through RDKit were rejected. The final set of SMILES strings was used to generate IUPAC names using the OpenEye Lexichem software 2.8.1. Several factors, including consistency with PubChem, influenced this decision: PubChem employs OpenEye Lexichem to generate IUPAC names. Using the same software ensured consistency with a major reference in the field. Also, OpenEye offers academic licenses, allowing us to use the software for scientific purposes without prohibitive costs.

The resulting dataset contains 883,897,289 SMILES, along with their corresponding IUPAC names. This dataset was then combined with the PubChem dataset from Experiment 2. SMILES strings with lengths above 600 characters were removed from the dataset for being less frequent in the dataset, resulting in a total of 999,637,326 molecules. The SMILES strings were tokenised as described previously, and the IUPAC names were split character-by-character. The resulting tokens included 132 SMILES tokens and 76 unique IUPAC name tokens. The maximum length of the SMILES input was set to 600 characters. In comparison, the maximum length of the IUPAC names was set to 700 characters, and after training for predictions, nearly 1,000 characters were allowed.

The SMILES to IUPAC and the IUPAC to SMILES models were trained using the same dataset.

### Testing datasets

#### Experiment 1—Impact of dataset size and tokenisation on model performance

The resulting six models were tested on three testing datasets: one with 250,000 molecules, one with 972,817 molecules, and another with 1 million molecules. To obtain the same token space as the 10 million training dataset, the test dataset has been reduced from 1 million molecules to 972,817 molecules for testing the model trained on the 10 million molecules. The slight variations in test dataset sizes across tokenisation methods are due to maintaining consistent token space and maximum length constraints relative to the training datasets. This approach ensures a fair comparison between models trained on different dataset sizes and tokenisation methods.

The test datasets were split into word splits, and character splits, resulting in three more testing datasets. Six combinations of test datasets were generated in total. Table 2 provides a comprehensive summary of the test datasets corresponding to the training datasets. For more information, please refer to the appendix Tokenisation.

**Table 2:** Test datasets compared to training datasets

	IUPAC name character split			IUPAC name word split		
Train data size	1 million	10 million	50 million	1 million	10 million	50 million
Test data size	265,332	972,817	1,024,000	288,152	990,080	1,024,000

### Experimental design considerations

The varying train: test ratios across experiments were a result of computational constraints in processing large datasets. While consistent ratios and multiple experimental runs would be ideal, these were not possible because of the significant computational resources required for the MaxMin algorithm and TPU training. Nevertheless, large test sets (> 250,000 molecules) were maintained across all experiments to ensure robust evaluations. Strict separation between training and test data was maintained throughout the study to preserve evaluation quality. These limitations are acknowledged, and more standardised approaches will be pursued in future work as resources permit.

Our experimental design focused on demonstrating the model’s ability to handle increased complexity with larger datasets, rather than isolating the impact of individual parameters such as token count of maximum length individually. We acknowledge that this approach doesn’t provide insights into the individual impact of each parameter, which could be a valuable direction for future work. The MaxMin algorithm was used for dataset selection of up to 100 million compounds but was not feasible for the billion-compound dataset due to computational constraints. So for experiment 3 the test dataset generated from experiment 2 is utilised.

#### Experiment 2—Training with increased data and longer sequences

As mentioned before, a test dataset of 1 million data points was selected from the downloaded data set from PubChem. After filtering this set to match the maximum lengths of IUPAC names and SMILES, 834,774 molecules remained.

#### Experiment 3—Large-scale training and generalization analysis

In experiment 3, the dataset from experiment 2 was used to test the model.

For external validation, we tested the final model using the ChEBI database molecules not included in the Training dataset from experiment 3. Lexichem software from OpenEye was used here to generate the IUPAC names for the molecules found on the ChEBI dataset. A diverse set of 1500 examples was selected from this dataset. SMILES with lengths exceeding 600 characters were removed, resulting in 1485 data points. The IUPAC names for these data points were generated using OpenEye Lexichem and then predicted using the STOUT models trained on datasets of 50 million, 100 million, and approximately 1 billion samples. To evaluate the model's performance on longer SMILES strings, we created an expanded test dataset combining molecules from ChEBI and ChEMBL34 databases. Since we specifically selected data not present in our training set, the number of longer SMILES strings obtained from ChEBI alone was limited. Therefore, we supplemented this with additional long-chain molecules from ChEMBL to ensure comprehensive testing across different molecular lengths. For this test, we used a total of 2,025 data points.

### TFRecord generation

It is recommended that TFRecords be used as a basic data structure to speed up the training process using TPUs. All STOUT training datasets were converted from string format to TFRecord files and saved in 100 MB chunks. This approach enhances the speed of reading TFRecords through the network. After generating the TFRecords, they were moved to Google Cloud Buckets in the same location where the TPUs were created.

### Model selection

In this work, the model implemented was from the 2017 publication “Attention is All You Need” by Vaswani et al. [35]. The transformer model implemented follows a contemporary architecture for sequence-to-sequence tasks, drawing on key elements from recent advances in deep learning. Here, the model features a stack of 4 transformer layers, each incorporating a multi-head attention mechanism with 8 heads and feed-forward networks with an internal dimension (dff) of 2048 and an embedding size (d_model) of 512, as detailed in the architecture proposed by Vaswani et al. A dropout rate of 0.1 is applied to prevent overfitting.

The optimisation process employs the Adam optimiser with a custom learning rate scheduler that adjusts based on the model dimensions, as suggested in the transformer model's original implementation. A custom schedule function defines this learning rate schedule, dynamically modifying the learning rate throughout training. The loss is calculated using the Sparse Categorical Cross-Entropy loss function, with a mechanism to ignore padding tokens. This is achieved by applying a boolean mask that excludes padded elements from the loss computation, ensuring the model does not learn from these irrelevant input parts. Key metrics for evaluation include training loss, which is tracked using a Keras Mean metric, and training accuracy, which is measured using Sparse Categorical Accuracy. These metrics provide critical insights into the model's performance and guide adjustments during the training process.

The selection of these parameters (4 transformer layers, 8 attention heads, internal dimension of 2048, embedding size of 512, dropout rate of 0.1) was the result of multiple iterations of training and testing [36] (data not shown). This iterative process ensured optimal performance for the task while adhering to the principles of the original transformer architecture.

The transformer is initialised with specific parameters for input and output vocabulary sizes and maximum sequence lengths for both input and target sequences. This comprehensive configuration and detailed implementation demonstrate the model's capability to handle complex language translation tasks, aligning with the current popular methodologies in neural machine translation.

### Model training

All STOUT models were completely trained on the Google Cloud Platform (GCP) using TPU V4 VMs. Training larger models requires considerable time, and using TPUs helps accelerate the training process. GCP offers a variety of TPUs; in this work, the models were trained on a TPU VM pod slice with 128 nodes. To enable training on TPU devices, all datasets were converted to TFRecord files.

TPUs are specialised hardware accelerators designed to optimise machine learning workloads, specifically neural network training. The choice of TPU V4 and the configuration used in this study are among the most advanced, offering high computational power and efficiency, which are crucial for handling the complexity and scale of modern deep learning models. Converting datasets to the TFRecord format ensures efficient data loading and preprocessing, further enhancing the training performance.

The models were trained using a batch size of 96 per node and an overall batch size of 6144. Training scripts and models were written in Python 3 with Keras and TensorFlow 2.15.0, and the training was done using TensorFlow 2.15.0-pjrt.

### Testing metrics

#### SMILES to IUPAC name translation testing

All model test results were evaluated for identical SMILES to IUPAC translation predictions. The predicted IUPAC name string was compared with the original string to determine whether it matched. If the predicted string was not identical to the original, it was rejected as not identical.

Only in Experiment 3 were all the generated IUPAC names retranslated back into SMILES using OPSIN 2.8.0. OPSIN was chosen because it systematically parses IUPAC names with a defined set of rules. The retranslated SMILES strings were compared with the original SMILES strings to determine the similarity between the original and predicted structures. Identical structures and the Tanimoto similarity indices (using PubChem fingerprints) were evaluated.

The overall average of these values was used for the final assessment of a model's accuracy. The average Tanimoto similarity was calculated using PubChem fingerprints as implemented in the CDK version 2.9.0. For each molecule pair (original and predicted), we computed the Tanimoto coefficient between their fingerprints. The Average Tanimoto was then calculated as the mean of these coefficients across all molecule pairs in the test set. When calculating the average Tanimoto similarity, predictions that failed to generate valid chemical structures were assigned a value of 0 and included in the calculation. This approach provides a more conservative estimate of model performance by accounting for both successful and failed predictions.

#### IUPAC name to SMILES translation testing

The IUPAC to SMILES test results were evaluated by performing a one-to-one string match between the original and predicted SMILES to determine how similar the predicted structures were to the original structures. To assess the structural similarity, a Tanimoto similarity index calculation (using PubChem fingerprints) was performed between the original and predicted SMILES strings.

The overall average of the prediction accuracy and the Tanimoto similarity were considered for evaluating a model's accuracy.

### Web app implementation

The web service implementation uses Vue.JS [37] for the front end and FastAPI [38] for the back end. FastAPI, chosen for its speed and efficiency, forms the backbone of the API, enabling the creation of advanced, scalable RESTful endpoints and helping to integrate with Python functions. This choice facilitates rapid development and deployment of the backend services while ensuring high performance and reliability. The front end and the back end are containerised using Docker.

Containerisation with Docker ensures consistent application execution across different environments, simplifying dependency management and streamlining the deployment process. Semantic versioning principles are applied to track changes in the codebase and toolkit versions, ensuring clear and manageable updates and backward compatibility.

The web app provides functionality to convert SMILES to IUPAC names, including a bulk translation option for multiple SMILES strings. It allows the retranslation of generated IUPAC names back to SMILES using OPSIN for verification. The app also offers options to translate IUPAC names to SMILES using either STOUT or OPSIN and to search for IUPAC names in PubChem using pubchempy. Additionally, a new feature enables the translation of chemical structure images to IUPAC names using DECIMER [19, 39].

## Results and discussion

The development of STOUT V2 demonstrates the transformer models' capacity and the fact that neural networks can accurately perform the non-trivial task of converting SMILES to IUPAC names. This work was solely possible due to the access of much-established rule-based systems like OpenEye’s Lexichem software. Our primary goal of this work is to make this work available to a wider audience, including those without a programming background, to simplify their IUPAC naming tasks. The development process was centred on creating an accurate, tool that leverages the capabilities of the transformer-based models rather than competing with any existing rule-based systems.

### Ensuring consistency in IUPAC name generation

In this work, it was ensured that consistent IUPAC names for unique chemical compounds were generated. To ensure this consistency, a preprocessing step using RDKit (version 2023.03.1) is implemented in the preprocessing pipeline. When a SMILES string is input by a user, it is first processed through RDKit to generate a canonicalised, isomeric, and kekulized SMILES string. This standardised SMILES string is then used as an input for STOUT, ensuring that for any given molecule, the same unique RDKit-based SMILES string is always generated, leading to consistent IUPAC name output. It should be noted that while valid and consistent IUPAC names are aimed to be generated by STOUT, Preferred IUPAC Names (PINs) [1] as defined by IUPAC are not necessarily produced. Instead, the naming conventions of Lexichem (version TK 2.8.1) are replicated by STOUT, which partially supports PINs but may generate alternative valid IUPAC names in some cases.

In the training and testing datasets, it has been ensured that each unique compound is associated with only one IUPAC name by applying consistent RDKit preprocessing and Lexichem name generation across all data. The risk of synonyms or inconsistent naming within the datasets is minimized by this approach. The reverse translation of STOUT-generated names to SMILES using OPSIN (version 2.7.0) in Experiment 3 was primarily conducted to validate the chemical validity of the generated names, rather than to address naming inconsistencies. In future, complex topics such as the intricacies of chemical naming, including the handling of synonyms and the relationship to PINs could be explored more in detail.

### Analysis of the results

**Fig. 3:**
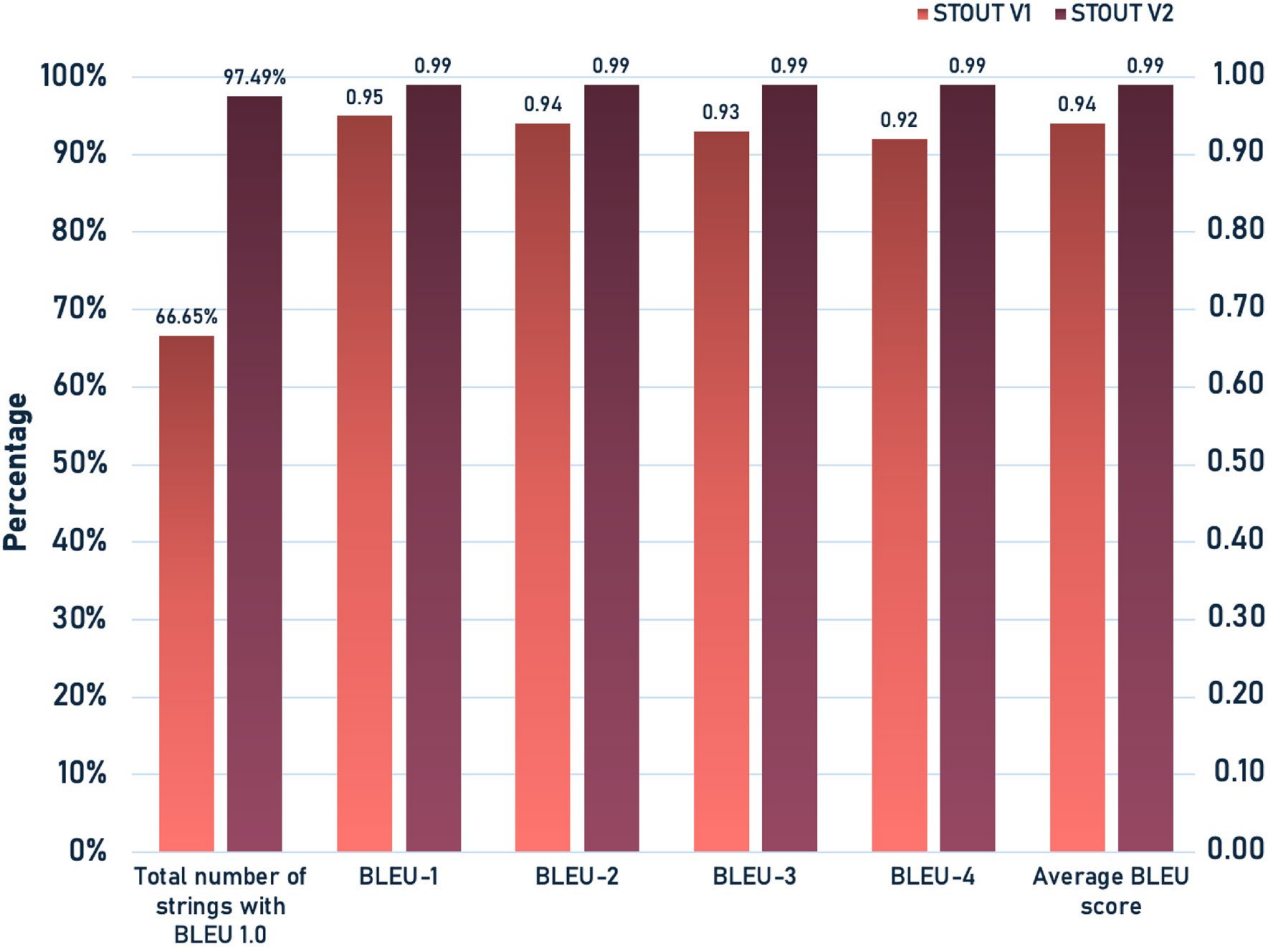
Comparison of BLEU Scores Between STOUT V1 and STOUT V2 Models

To evaluate how STOUT V2 performs compared to STOUT V1, a comparative analysis between STOUT V2 and V1 was conducted using the BLEU (Bilingual Evaluation Understudy) scoring metric. BLEU is a metric for evaluating the quality of machine-translated text by comparing it with reference strings (ground truth), primarily focusing on the precision of matching n-grams [40]. Both models were trained on the same 60 million molecule dataset originally employed for STOUT V1 [26], and testing was performed on the identical test dataset of 2.2 million molecules to ensure a fair comparison. The performance comparison between STOUT V1 and V2 across various BLEU metrics is illustrated in Fig. 3. Significant improvement by STOUT V2 over V1 is demonstrated in all measured categories. An increase in the average BLEU score from 0.94 for V1 to 0.99 for V2 was observed, indicating improved translation quality. Furthermore, perfect BLEU scores (1.0) were achieved by STOUT V2 for a substantially higher percentage of strings (97.49%) compared to V1 (66.65%). The performance of STOUT V2 is further observed consistently through the scores across BLEU-1 through BLEU-4, suggesting improved performance in capturing both individual tokens and longer n-gram sequences. Based on this comprehensive analysis, it can be concluded that STOUT V2 represents a substantial improvement over STOUT V1 in translating SMILES to IUPAC names. STOUT V1 was trained using TPU V3-8 nodes with TensorFlow 2.3.0. In contrast, STOUT V2 was trained entirely on a TPU V4-128 pod slice using TensorFlow 2.15.0-pjrt.

#### Experiment 1—Impact of dataset size and tokenisation on model performance

In this experiment, a series of models were trained with SMILES as inputs and IUPAC names as outputs, progressively increasing the dataset size.

The IUPAC names were divided into tokens using two separate methods: word-wise splitting and character-wise splitting (see appendix). Three models were trained for each combination based on 1 million, 10 million, and 50 million training data points selected from PubChem as seen in Table 2.

In this study, all models were tested against the respective test datasets discussed in the dataset section. Each model was compared on its ability to predict IUPAC names based only on one-to-one string matches since we are primarily interested in a model that can produce more accurate IUPAC names overall.

**Fig. 4:**
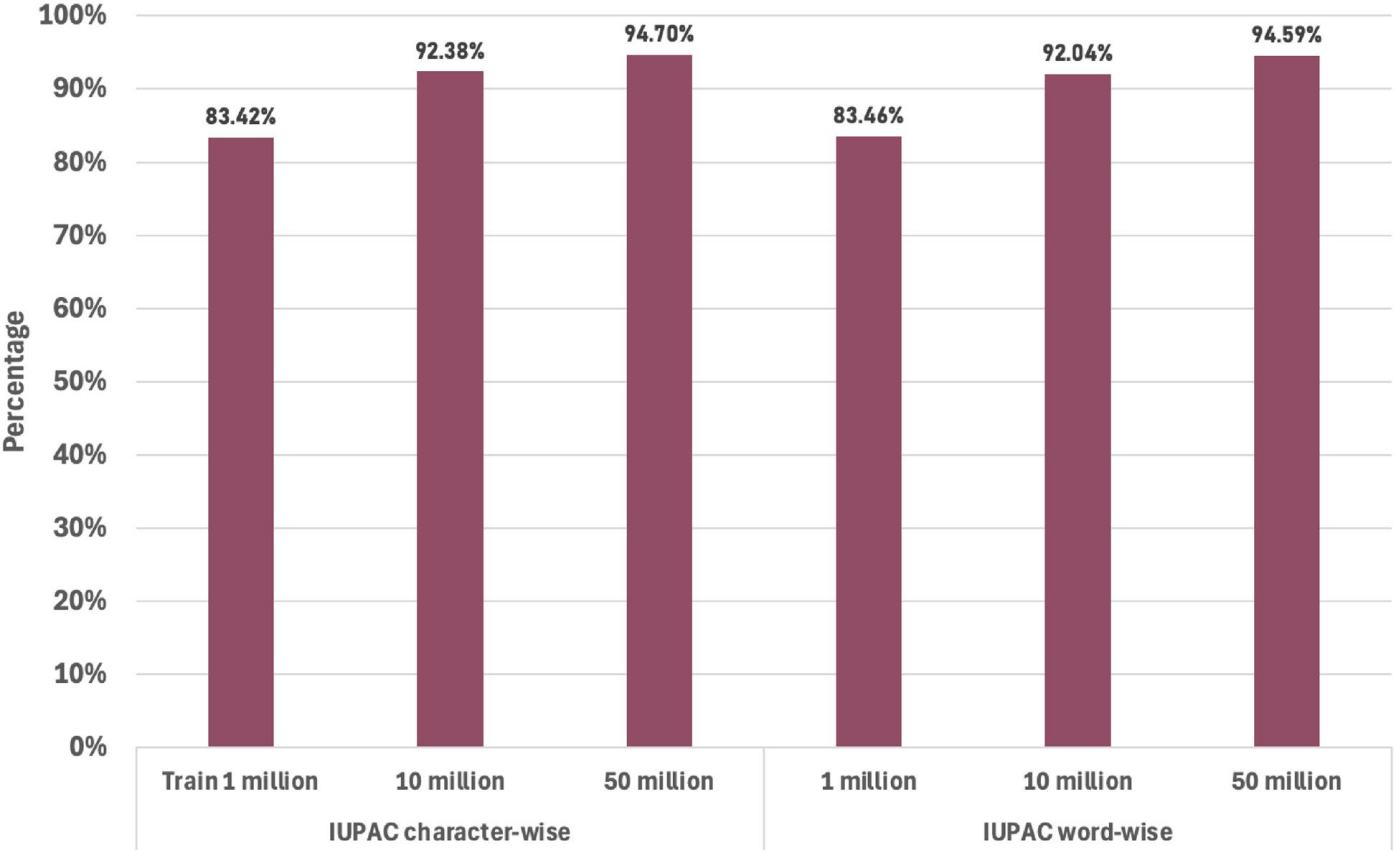
Comparison of SMILES representation performance using IUPAC character-wise and word-wise split approaches across different dataset sizes (1 Million, 10 Million, and 50 Million molecules)

According to Fig. 4, the performance of SMILES models improves with increasing dataset size, while the increment with unique tokens and maximum length has little effect on performance. This trend of improved performance correlating with larger datasets is observed across the results.

When examining IUPAC names tokenised by rules versus tokenised by character, the character-level tokenisation demonstrates better performance in models trained with more than 1 million data points. Overall, the IUPAC split by characters performs better when used as output using SMILES as input.

Character-level tokenisation showed a slight performance advantage and offered practical benefits such as implementation simplicity and reduced dependence on complex rule sets or third-party packages for tokenisation. It also results in a smaller token set, allowing for training with larger batches, which leads to easier model convergence in fewer epochs and, consequently, faster overall training. Also, as shown in Table 1, for the same batch size, the character split trains, on average more than 15% faster than the word split.

#### Experiment 2—Training with increased data and longer sequences

From Experiment 1, it was clear to use a Transformer model with SMILES as Input and IUPAC names character-by-character as output. With this information, a bigger dataset obtained from PubChem was used to train the same model to understand the performance gain and to see whether there is an impact on the performance by doubling the input and output maximum lengths of the strings.

The model was tested on 834,774 data points, and it was observed that it could produce 89.86% of accurate SMILES to IUPAC name translation when checked by a one-to-one string match. This reduction of accuracy was observed due to long complex names that were introduced, and the longer string prediction was also made to create errors.

Using the same dataset, when an IUPAC name-to-SMILES model was trained, the model performed with an accuracy of 94.46%, a Valid SMILES prediction of 99.54%, a Tanimoto 1.0 count of 97.47%, and an average Tanimoto of 0.99, indicating robust performance and high-quality predictions.

#### Experiment 3—Large-scale training and generalisation analysis

This experiment aimed to investigate the impact of significantly increasing the training dataset size on the performance of a transformer model for IUPAC name translation. Expanding the dataset tenfold provided the model with a substantially larger number of examples, hypothesising that this would lead to improved learning of IUPAC name translation patterns and better overall generalisation.

The model for this study was trained on a dataset of nearly one billion data points, as described in the methods section. The training was carried out on a TPU v4 VM with a 256-node pod slice, with each epoch taking an average of 15 h and 2 min. A second model was also developed to perform the reverse task: IUPAC names were translated back into SMILES strings. This model used the same dataset but with the input and output string representations reversed. The approach was used to explore bidirectional conversion between chemical structures and their standardised names.

The model was then evaluated using the test dataset from Experiment 2, yielding a performance of 83.52% for exact string match in SMILES to IUPAC name translation.

For IUPAC to SMILES performance on the same test set, the model achieved a test accuracy of 90.46% with a Valid SMILES prediction of 99.1%, Tanimoto 1.0 count of 95.11% and a Tanimoto average of 0.99.

This observed decrease in accuracies compared to Experiment 2 could be due to the larger and more diverse dataset. While this approach enhances generalisability, it can also lead to a slight decrease in performance on any single test set. The exposure to a broader range of examples increased the likelihood of generating errors. Adding additional examples from sources other than PubChem may have caused a data distribution shift [41, 42]. This shift could have introduced training examples that differ significantly from those in the test dataset, potentially leading to reduced model performance on the original test set.

By increasing the maximum length of IUPAC names in training and predictions, the model can learn from longer complex names, but it also increases prediction errors.

A benchmark study using the ChEBI dataset was conducted to understand whether the model improved overall. This experiment checked the models' ability to generalise and handle previously unseen data, thus providing insights into their real-world applicability.

This test assessed the model's ability to generate correct IUPAC names by comparing the predicted and original names using exact string matching.

**Fig. 5:**
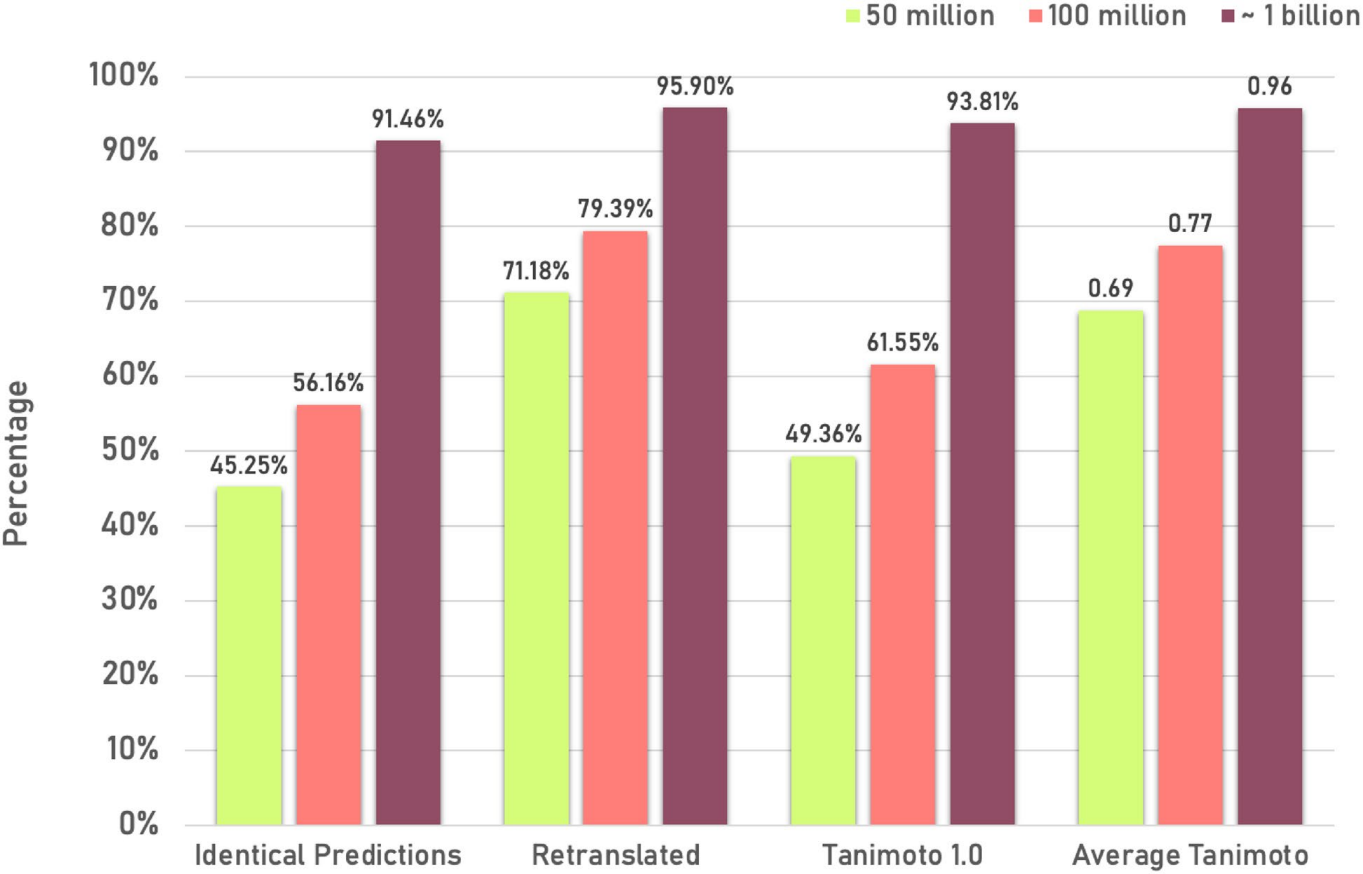
Performance of the SMILES to IUPAC name translation model with increasing dataset size. Comparing the identical predictions, Percentage of IUPAC names retranslation, Tanimoto 1.0 percentage and Average Tanimoto for models

To ensure that the predicted IUPAC names represent a valid chemical structure, a two-step verification process was implemented. The predicted IUPAC names were reverse-translated back into SMILES notation using OPSIN, and the resulting SMILES strings were then compared to the original input SMILES strings to verify how similar the original and the retranslated structures are using Tanimoto similarity using PubChem fingerprints incorporated in Chemistry Development Kit (CDK) [43, 44]. See Fig. 5 for more details.

Looking at the results in Fig. 5, the identical predictions and the retranslation accuracy increase with more training data, implying that the model's predicted IUPAC names become more consistent and fluent with more extensive training. Tanimoto 1.0 and Average Tanimoto also improve significantly with more training data, reflecting a higher quality in the model's predictions as it is trained on larger datasets. Overall, this suggests that increasing the training data improves the model's generalisability, and one could get better and more accurate IUPAC names.

To further understand the implications of the length of predicted IUPAC names, the ChEBI benchmark test in combination with SMILES from the ChEMBL34 dataset was utilised. The predictions were categorised into 10 groups according to the input SMILES length, and the prediction quality was then analysed in detail.

**Fig. 6:**
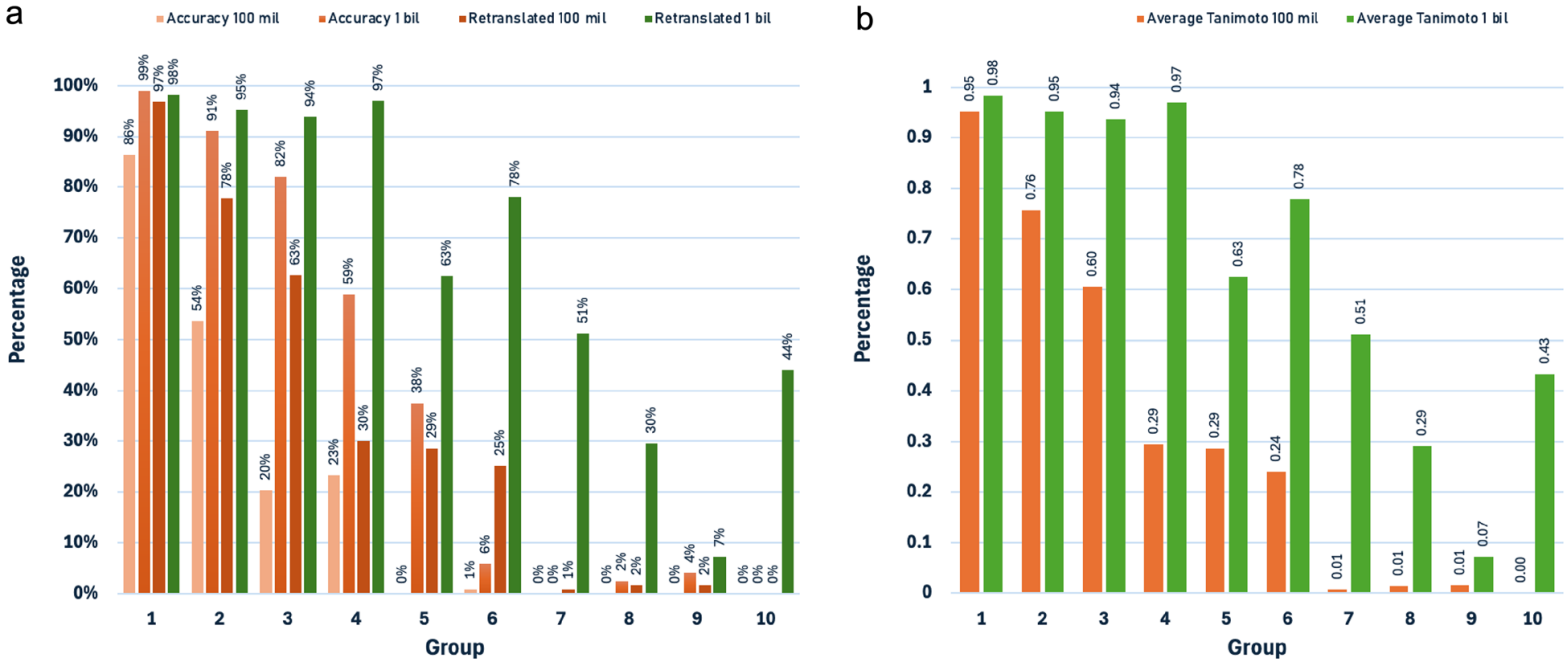
**a** IUPAC name prediction accuracy, retranslation quality, and (**b**) Average Tanimoto similarities for compounds with SMILES strings ranging from 0–600 characters, comparing performance between 100 million and 1 billion compound datasets across 10 length groups. SMILES string length groups (1–10): 0–60, 61–120, 121–180, 181–240, 241–300, 301–360, 361–420, 421–480, 481–540, and 541–600 characters

As shown in Fig. 6, both models show decreasing performance as SMILES length increases, but with notably different patterns. The model trained on 100 million data points shows a sharp decline in accuracy and retranslation success for longer SMILES strings, dropping to nearly zero accuracy for the longest sequences. In contrast, the model trained on 1 billion data points maintains significant prediction quality across accuracy, retranslation using OPSIN, and average Tanimoto similarity despite the increased complexity of the IUPAC names.

The retranslation fails for longer IUPAC names generated by the 100 million data points model due to errors such as missing spaces, incomplete predictions, incorrect group order, formatting issues, incorrect valency, and other errors in generated IUPAC names detected by OPSIN encountered during the retranslation of these names. These issues are reflected in Fig. 6b, where the average Tanimoto similarity for the 100 million model drops dramatically for longer molecules, while the 1 billion model maintains higher similarity scores across all length groups.

This analysis demonstrates that increasing the training dataset size not only improves the model's ability to handle longer, more complex SMILES strings but also results in more robust IUPAC name generation across different molecular lengths.

Similarly, the IUPAC to SMILES translation models, trained on 100 million and approximately 1 billion data points, were also compared using the ChEBI dataset. As seen in Table 5 the models trained on 100 million and 1 billion data points perform almost similarly. However, the model trained on 1 billion data points, which included IUPAC names longer than 600 characters, had to learn more complex IUPAC names. This complexity slightly reduced the model's performance compared to the 100 million data point model. In general, the performance of both models is comparable.

**Table 5:** Performance of IUPAC to SMILES models trained on increasing training data

Training data size	Valid SMILES	Identical predictions	Tanimoto 1.0	Average tanimoto
100 million	99.80%	96.97%	99.12%	0.99
~ 1 billion	99.60%	96.23%	99.06%	0.99

**Fig. 7:**
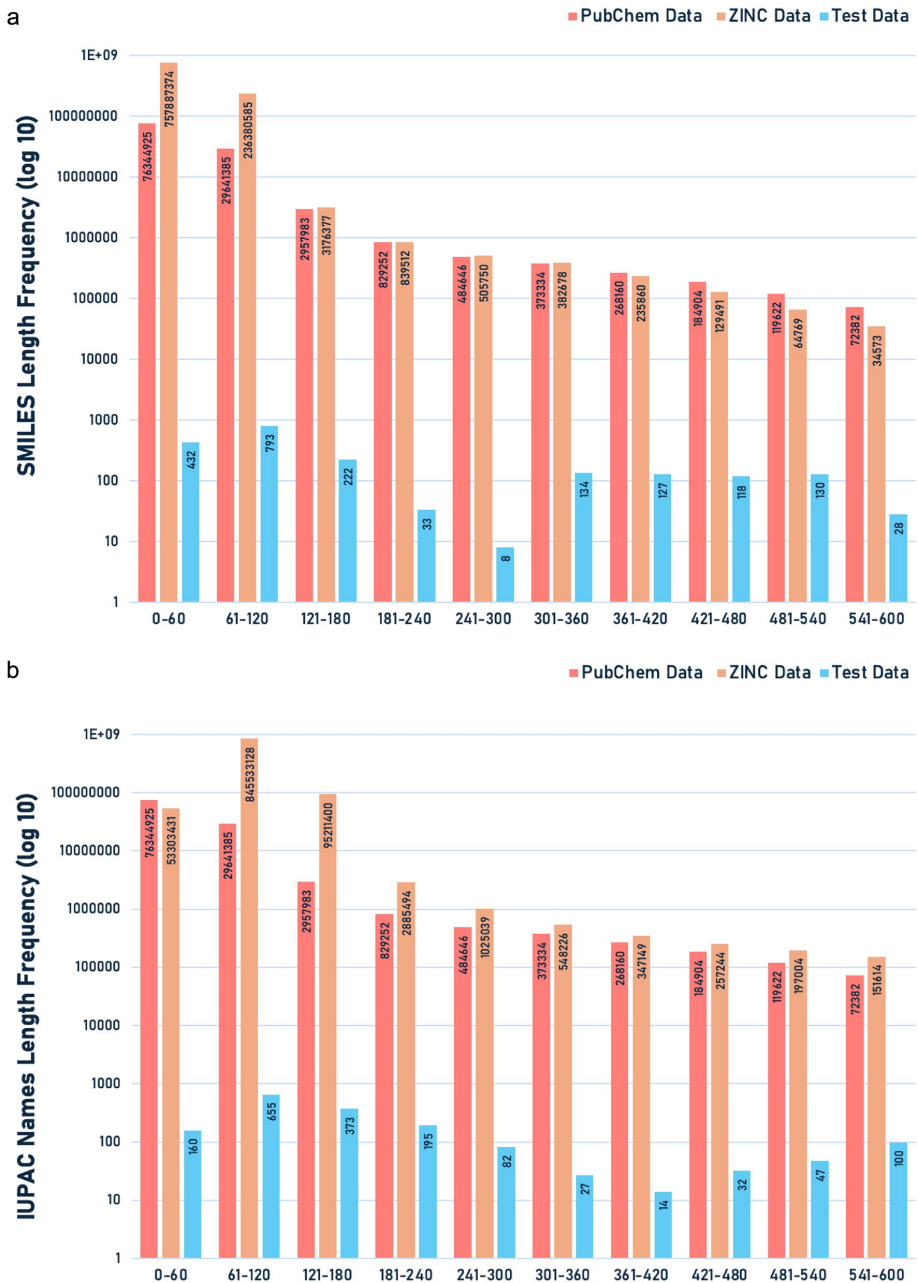
SMILES (**a**) and IUPAC (**b**) names Length Distribution Comparison: PubChem, ZINC, and the final test data

To contextualise the observed decrease in prediction quality for longer SMILES, we analysed the distribution of SMILES lengths in our training and the final test sets. Figure 7 below shows the percentage of SMILES (a) and IUPAC names (b) in each length group for both the 100 million and 1 billion compound datasets, as well as the final test set.

The underrepresentation of longer SMILES in our datasets likely explains the decreased performance on more complex structures. This imbalance reflects a common limitation in available open chemical databases, where compounds with longer SMILES are relatively scarce. As more comprehensive and well-curated datasets become available, future iterations of our model could potentially benefit from increased exposure to these more complex structures, potentially leading to improved performance across a wider range of molecular complexities.

In this work, when evaluating the accuracy of our model, we only considered AI-generated IUPAC names (AINs) that exactly matched the original, correct IUPAC names. Any AINs that differed from the correct names, even slightly, were counted as incorrect and excluded from the accuracy calculation. This strict approach was taken because, in practice, chemists require IUPAC names to be 100% accurate to be useful. Slightly different names are still considered incorrect.

It is important to note that potential limitations may arise from the differences between the datasets used. Despite consistent preprocessing across all datasets, slight variations between sources such as PubChem and ZINC might still exist. These differences, although minimal, could influence the model's performance and generalisability. The full extent of this impact could not be completely measured within the scope of this study. Future research could focus on a more detailed analysis of these dataset-specific characteristics and their effects on STOUT's output, which would provide valuable insights into the model's robustness across diverse chemical data sources.

To evaluate the model's ability to handle slightly different IUPAC names, names that did not match the original IUPAC name were retranslated using OPSIN, and the resulting structures were compared. A few examples are summarised in Table 6. In this analysis, we refer to the original Lexichem-generated IUPAC names as reference IUPAC names, while the model's generated names are referred to as AI-generated IUPAC Names (AINs). The primary goal is for AINs to match the reference IUPAC names exactly, though it is recognized that multiple valid IUPAC names can represent the same chemical structure. While Lexichem generates standardized IUPAC names following systematic rules, these names are not guaranteed to be Preferred IUPAC Names (PINs) as defined by IUPAC guidelines.

**Table 6:** Comparison of original structures and reference IUPAC names with OPSIN retranslated structures derived from AI-generated IUPAC Names (AINs), Illustrating Model Robustness and Alternative Naming. (1 billion model)

Original compound (reference | original structure)	STOUT-generated compound (AIN | OPSIN-translated structure)	Tanimoto similarity
**bis**[bis(2-methylpropyl)amino]-**bis**[ethyl(methyl)amino]azanium	**tris**[bis(2-methylpropyl)amino]-[ethyl(methyl)amino]azanium	1.0
5-[(11S,12S)-12-(aminomethyl)-**6**-fluoro-3-[[(2S,4R)-4-fluoro-1-methylpyrrolidin-2-yl]methoxy]-11-methyl-13-pent-3-ynyl-10-oxa-2,4,8,13-tetrazatricyclo[7.4.1.05,14]tetradeca-1,3,5(14),6,8-pentaen-7-yl]-2-fluoro-3-methyl-4-(trifluoromethyl)aniline	5-[(11S,12S)-12-(aminomethyl)-**8**-fluoro-3-[[(2S,4R)-4-fluoro-1-methylpyrrolidin-2-yl]methoxy]-11-methyl-13-pent-3-ynyl-10-oxa-2,4,6,13-tetrazatricyclo[7.4.1.05,14]tetradeca-1,3,5(14),6,8-pentaen-7-yl]-2-fluoro-3-methyl-4-(trifluoromethyl)aniline	0.96
[(**1R,5S**)-3-pentadecan-8-yl-3-azabicyclo[3.2.1]octan-8-yl]cyanamide	[(**1S,5R**)-3-pentadecan-8-yl-3-azabicyclo[3.2.1]octan-8-yl]cyanamide	1.0
(1R,3S,**5S**,6R)-5-[(hex-5-enoylamino)methyl]-6-methyl-N-[3-methyl-6-(trifluoromethyl)pyridin-2-yl]-2-azabicyclo[3.1.0]hexane-3-carboxamide	(1R,3S,**5R**,6R)-5-[(hex-5-enoylamino)methyl]-6-methyl-N-[3-methyl-6-(trifluoromethyl)pyridin-2-yl]-2-azabicyclo[3.1.0]hexane-3-carboxamide	1.0
6-[3-(1,3-benzothiazol-2-ylamino)-4-methyl-6,7-dihydro-5H-pyrido[2,3-c]pyridazin-8-yl]-3-[1-[[5-[2-[carboxymethyl(methyl)amino]ethoxy]-**3,7-dimethyl**-1-tetracyclo[5.3.1.03,9.05,9]undecanyl]methyl]-5-methylpyrazol-4-yl]pyridine-2-carboxylic acid	6-[3-(1,3-benzothiazol-2-ylamino)-4-methyl-6,7-dihydro-5H-pyrido[2,3-c]pyridazin-8-yl]-3-[1-[[3-[2-[carboxymethyl(methyl)amino]ethoxy]-**5,7-dimethyl**-1-tetracyclo[5.3.1.03,9.05,9]undecanyl]methyl]-5-methylpyrazol-4-yl]pyridine-2-carboxylic acid	0.99

To assess this, a two-step process is employed: first, SMILES representations are converted to AINs using the model (SMILES≥ AIN), and then these AINs are converted back into valid chemical structures using OPSIN (AIN≥ OPSIN SMILES). These OPSIN-translated structures are compared with the original structures using Tanimoto similarity. Cases, where AINs are not exactly equal to reference names but Tanimoto similarity = 1.0, are considered to indicate valid alternative IUPAC names. This approach allows for the evaluation of both the model’s precision in generating reference IUPAC names and its ability to produce valid alternative IUPAC names that correctly represent the intended chemical structure.

As shown in Table 6, the five examples picked from incorrectly predicted AI-generated IUPAC names, while incorrect, produce chemical structures similar or identical to those of the original chemical structures. This suggests the model's errors in name prediction may be minor in these cases. To quantify the extent of this phenomenon across our test dataset, we comprehensively analysed all non-identical predicted IUPAC names. We converted these names to SMILES representations using OPSIN and calculated the Tanimoto similarity coefficient between the predicted and original structures. Of the predictions that generated non-identical IUPAC names compared to the original compounds, 30.7% (representing 2.6% of the total test dataset) could not be converted into valid chemical structures. Further analyses were conducted on the remaining 69.3% of predictions with non-identical IUPAC names but valid structural conversions. These analyses revealed an average Tanimoto similarity coefficient of 0.68, indicating moderate to high structural similarity despite the naming variations. The observed decrease in average Tanimoto similarity can be attributed to predictions that failed to generate valid chemical structures. Table 7 presents the five examples with the lowest Tanimoto similarity indices.

**Table 7:** Comparison of Original Structures and their reference IUPAC Names with OPSIN-Retranslated Structures Generated from AI-Predicted IUPAC Names (AINs): Cases with Lowest Tanimoto Similarity Indices

Original compound (reference | original structure)	STOUT-generated compound (AIN | OPSIN-translated Structure)	Tanimoto similarity
tert-butyl (7S,8R)-12-chloro-13-fluoro-16-methylsulfanyl-8-(trifluoromethyl)-9-oxa-2,5,11,15,17-pentazatetracyclo[8.7.1.02,7.014,18]octadeca-1(17),10,12,14(18),15-pentaene-5-carboxylate	tert-butyl (7S,8R)-12-chloro-11-fluoro-15-methylsulfanyl-8-(trifluoromethyl)-9-oxa-2,5,13,14,16-pentazatetracyclo[8.7.1.02,7.014,18]octadeca-1(17),10,12,15-tetraene-5-carboxylate	0.45
tert-butyl (8S,9S)-13-[3-[bis[(4-methoxyphenyl)methyl]amino]-2-fluoro-5-methyl-6-(trifluoromethyl)phenyl]-14-fluoro-4,9-dimethyl-17-methylsulfanyl-10-oxa-2,5,7,12,16,18-hexazapentacyclo[9.7.1.02,8.06,8.015,19]nonadeca-1(18),11,13,15(19),16-pentaene-5-carboxylate	tert-butyl (1S,2S)-13-[3-[bis[(4-methoxyphenyl)methyl]amino]-2-fluoro-5-methyl-6-(trifluoromethyl)phenyl]-12-fluoro-2,7-dimethyl-17-methylsulfanyl-3-oxa-5,8,10,14,16,18-hexazapentacyclo[9.7.1.01,9.04,9.015,19]nonadeca-11(19),12,14,16-tetraene-5-carboxylate	0.84
(1R,23R,26S)-17-acetyl-N-(6-bromo-3-methylpyridin-2-yl)-4,21-dioxo-27-thia-3,14,18,19,22-pentazapentacyclo[20.2.2.113,16.01,23.015,19]heptacosa-13,17-diene-26-carboxamide	(1R,22R,25S)-16-acetyl-N-(6-bromo-3-methylpyridin-2-yl)-4,20-dioxo-27-thia-3,14,15,19,23-pentazapentacyclo[21.2.2.111,14.01,22.013,17]octacosa-11,15-diene-25-carboxamide	0.88
3-[(1S,5R,6R,9R,14S,17R)-7-(6-aminopurin-9-yl)-6-fluoro-3,17-dihydroxy-12-oxo-12-sulfanyl-3-sulfanylidene-2,4,8,11,13-pentaoxa-3λ5,12λ5-diphosphatricyclo[12.2.1.05,9]heptadecan-15-yl]-6H-imidazo[4,5-b]pyridin-7-one	3-[(1S,6R,7R,10R,15S,18R)-8-(6-aminopurin-9-yl)-7-fluoro-3,18-dihydroxy-12-oxo-12-sulfanyl-3-sulfanylidene-2,4,9,11,13-pentaoxa-3λ5,12λ5-diphosphatricyclo[13.2.1.06,10]octadecan-16-yl]-6H-imidazo[4,5-b]pyridin-7-one	0.89
4-[3-[(3,3-difluoro-1-bicyclo[3.2.0]heptanyl)methoxy]-6-fluoro-12,14-dimethyl-10-oxa-2,4,8,11,14-pentazatricyclo[7.5.1.05,15]pentadeca-1,3,5(15),6,8-pentaen-7-yl]-5-ethynylnaphthalen-2-ol	4-[7-[(3,3-difluoro-1-bicyclo[3.2.0]heptanyl)methoxy]-8-fluoro-12,14-dimethyl-10-oxa-2,4,6,11,14-pentazatricyclo[7.5.1.05,15]pentadeca-1,3,5(15),6,8-pentaen-3-yl]-5-ethynylnaphthalen-2-ol	0.90

Our primary goal is to generate the exact IUPAC name for a given molecule, the same as the reference IUPAC name. The additional calculation of Tanimoto similarity values attempts to examine the cases where the predicted names differ from the original ones to see if the model begins to “understand the underlying chemistry”. Though widely used in cheminformatics, these similarity scores have limitations, as they rely on finite molecular fingerprints that may not capture all structural features. Our results show that structurally distinct molecules can sometimes achieve perfect similarity scores (Tanimoto = 1.0). However, since we primarily evaluate performance through exact string matching of IUPAC names, these limitations do not affect our main conclusions about the model’s accuracy.

Despite their non-identical IUPAC names, when these predictions were successfully converted into valid chemical structures, they demonstrated high structural similarity to the original compounds. Visual inspection of the depicted structures reveals that these naming discrepancies are readily identifiable and could be corrected by a trained chemist.

Despite these challenges, the model often generates names that, when translated back to structures, show high structural similarity to the original compounds. However, it’s crucial to note that this similarity does not guarantee chemical equivalence or safety, especially in contexts like drug discovery where precise naming is critical. While further refinement is needed, these results demonstrate the model's potential value in assisting with compound nomenclature tasks.

Some names failed to retranslate completely due to errors in the predicted names and limitations within OPSIN. Examples of these errors are listed in Table 8.

**Table 8:** Examples of OPSIN retranslation errors

Original compound (reference | original structure)	STOUT-generated compound (AIN | OPSIN-translated structure)	Problems identified by OPSIN with generated names
tert-butyl 12-(2-aminoquinolin-8-yl)-13-fluoro-16-methoxy-8-methyl-9-oxa-2,5,11,15,17-pentazatricyclo[8.7.1.014,18]octadeca-1(17),10,12,14(18),15-pentaene-5-carboxylate	tert-butyl 15-(2-aminoquinolin-8-yl)-14-fluoro-17-methoxy-11-methyl-10-oxa-2,5,8,16,18-pentazatricyclo[7.7.1.013,17]heptadeca-1(16),9(17),13(17),14-tetraene-5-carboxylate	Could not find the atom with locant 18
tert-butyl (8S)-12-chloro-13-fluoro-16-methoxy-6,8-dimethyl-9-oxa-2,5,11,15,17-pentazatricyclo[8.7.1.014,18]octadeca-1(17),10,12,14(18),15-pentaene-5-carboxylate	tert-butyl (3S)-16-chloro-15-fluoro-13-methoxy-3,5-dimethyl-2-oxa-6,9,11,12,14-pentazatricyclo[8.6.1.015,17]heptadeca-1(16),10,12,14-tetraene-6-carboxylate	Atom is in unphysical valency state! Element: C valency: 5
N'-[[methyl-[1-(6-spiro[1,2-dihydrofluorene-9,9'-6,7-dihydroxanthene]-3'-ylpyridin-3-yl)ethyl]amino]-phenylmethyl]benzenecarboximidamide	N'-[[methyl-[1-(6-spiro[1,2-dihydrotriphenylene-9,9'-6,7-dihydroxanthene]-3'-ylpyridin-3-yl)ethyl]amino]-phenylmethyl]benzenecarboximidamide	Failed to assign all double bonds! (Check that indicated hydrogens have been appropriately specified)
(7S,10S,13S)-N-[(1S)-1-cyclopropylethyl]-10-(2-morpholin-4-ylethyl)-9,12-dioxo-13-(2-oxopyrrolidin-1-yl)-2-oxa-8,11-diazabicyclo[13.3.1]nonadeca-1(18),15(19),16-triene-7-carboxamide	(7S,10S,13S)-N-[(1S)-1-cyclopropylethyl]-13-(2-morpholin-4-ylethyl)-9,12-dioxo-16-(2-oxopyrrolidin-1-yl)-2-oxa-8,11-diazabicyclo[16.3.1]docosa-1(21),18(22),19-triene-7-carboxamide	Could not find atom that: < stereoChemistry locant = "10" type = "RorS" value = "S" stereoGroup = "Abs" > 10S < /stereoChemistry > appeared to be referring to
(4R,6R)-6-[(1R)-1-[[2-[amino(methanimidoyl)amino]acetyl]amino]ethyl]-4-methyl-7-oxo-3-[(3S,5S)-5-[3-(1,2,4-triazol-4-yl)azetidine-1-carbonyl]pyrrolidin-3-yl]sulfanyl-1-azabicyclo[3.2.0]hept-2-ene-2-carboxylic acid	4R,6R)-4-[(1R)-1-[[2-[amino(methanimidoyl)amino]acetyl]amino]ethyl]-6-methyl-2-oxo-3-[(3S,5S)-5-[3-(1,2,4-triazol-4-yl)azetidine-1-carbonyl]piperidin-3-yl]sulfanyl-1-azabicyclo[3.2.0]hept-3-ene-8-carboxylic acid	Suffix: imido does not apply to the group it was associated with (type: standardGroup) according to suffixApplicability.xml

Table 8 reveals several issues in automated chemical nomenclature using deep learning. While many predicted names were successfully retranslated, some failed due to errors in the generated names and limitations within OPSIN. The errors mentioned above indicate the complexities of automated IUPAC name generation and interpretation, particularly for intricate organic molecules. Despite these challenges, the model could generate structurally similar names in many cases. However, the results underscore the continued importance of expert verification in deep learning-based chemical nomenclature tasks and point to specific areas for improvement in name-generation models and parsing software like OPSIN.

Below are a few examples of valid IUPAC names that OPSIN could not retranslate, along with the corresponding OPSIN errors:

- (1 s,4 s)-4-(chlorooxy)cyclohexyl hypofluorite.

*Error: cannot parse this due to known limitation in OPSIN's stereochemistry support.*

- tert-butyl 4-[7-chloro-8-fluoro-2-[[(2R,8S)-2-fluoro-1,2,3,5,6,7-hexahydropyrrolizin-8-yl]methoxy]-5-methoxypyrido[4,3-d]pyrimidin-4-yl]piperazine-1-carboxylate.

*Error: could not find the atom with locant 8.*

- bis[(2S)-2-amino-5-(carbamoylamino)pentanoyl] (2R)-2-acetyl-2-aminopentanedioate;(2R)-2,5-diaminopentanoic acid.

*Error: IUPAC names for disconnected molecules cannot be parsed. Removing the ";" allows parsing of the name.*

- (8S)-8-(ethoxymethyl)-3-[[5-(trifluoromethyl)pyridazin-3-yl]oxymethyl]-1,2,3,5,6,7-hexahydropyrrolizine.

*Error: cannot find in scope fragment with an atom with locant 8.*

### STOUT web application

**Fig. 8:**
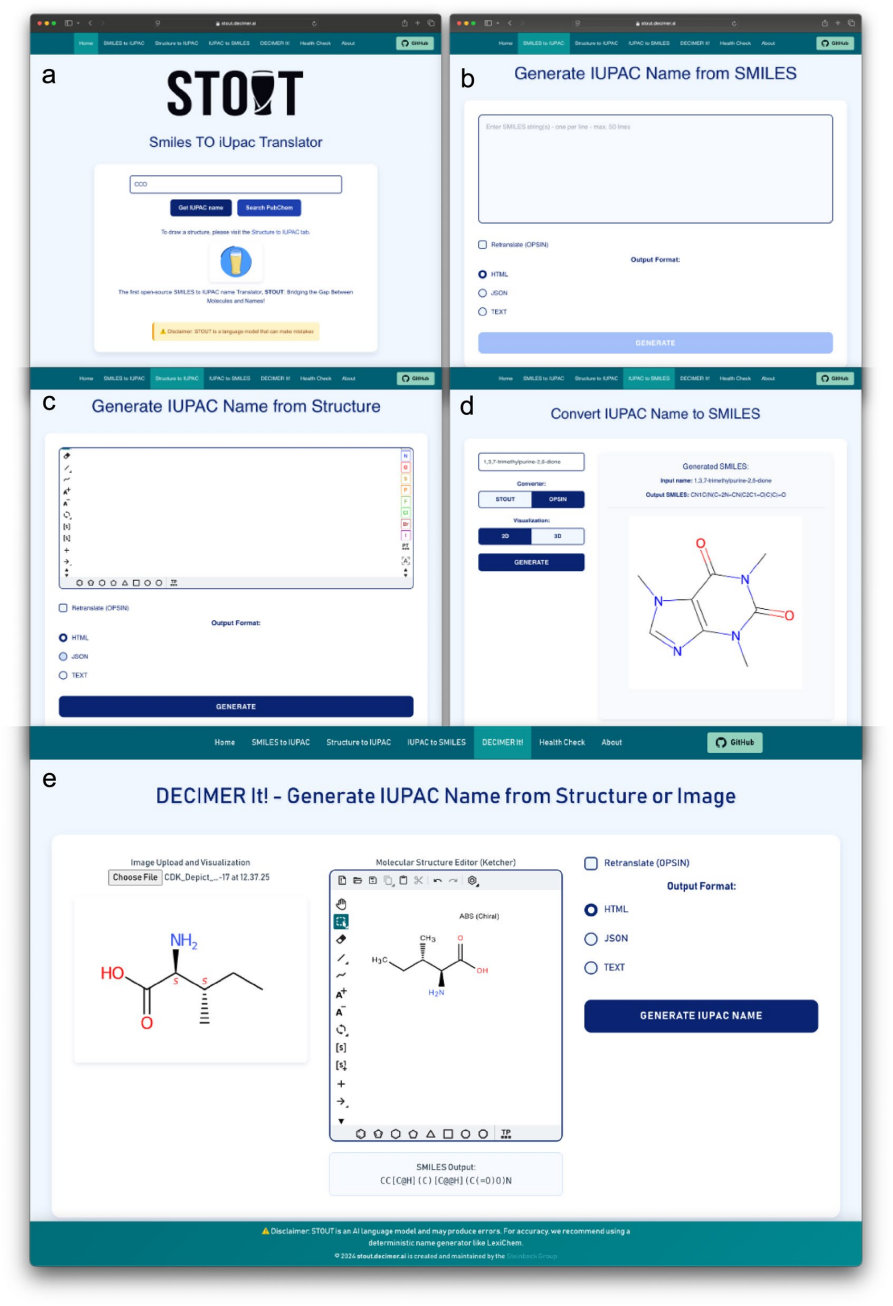
The STOUT web application interface, featuring a home page for single SMILES input (**a**), batch processing for up to 50 SMILES strings (**b**), the Ketcher editor for drawing structures (**c**), the IUPAC to SMILES translation with structure depiction using CDK (**d**) and a DECIMER to SMILES for translating a chemical structure image to IUPAC name (**e**)

This work also includes a web application as a demo as seen in Fig. 8 designed to support the automated naming of chemical structures for chemists or chemical database curators. The Web App, leveraging the STOUT models, allows users to input SMILES strings of the chemical compounds and receive IUPAC names. It also provides a feature to check whether the compound is already catalogued in PubChem, displaying the corresponding IUPAC name, if available, and retrieved directly from PubChem.

The Web App supports bulk submission of up to 50 SMILES strings, facilitating the generation of IUPAC names for multiple compounds simultaneously. These generated names can be retranslated using OPSIN and visualised using CDK at the backend, enabling users to compare the original and predicted structures. The results can be downloaded in HTML, JSON, or text formats.

It also includes a Ketcher [45] window for drawing chemical compounds and obtaining their IUPAC names. For IUPAC names to SMILES conversion, users can choose to utilise either STOUT or OPSIN, with the Web App depicting the resulting structure for visual analysis of the predictions. This web application is completely containerised, so those who do not want to use the web application available online can spin up their web application locally using the Docker container.

## Conclusion

This work presents STOUT V2, an improved successor for SMILES to IUPAC name translator. This work represents a significant advancement in automated deep learning-based IUPAC name generation. It was, however, only possible due to the availability of IUPAC names generated by deterministic naming algorithms, in our case, by OpenEye’s Lexichem software. A substantial improvement was achieved by leveraging the transformer-based models for deep learning.

In terms of tokenisation strategy, it was discovered that character-split tokenisation of IUPAC names yielded better overall accuracy compared to word-split tokenisation. These character-split models also demonstrated faster training times and utilised fewer unique tokens (66 vs 1,501 tokens for 50 million dataset).

We conducted large-scale training on a dataset of nearly one billion compounds to improve model generalisability. As shown in our results using the final test set, the model trained on about 1 billion compounds maintains prediction accuracy and high retranslation success rates even for longer SMILES strings, while the 100 million compound model's performance drops significantly with increasing molecular complexity. This suggests the increased training data enhanced the model’s ability to handle a wider range of chemical structures. Future work will focus on enhancing the model’s performance on longer molecular structures, particularly those exceeding 300 characters in SMILES strings. While our current model trained on nearly 1 billion compounds demonstrates good performance across various molecular lengths, targeted datasets including more complex structures could further refine the model's capabilities.

By expanding the maximum input length to 700 characters and extending the maximum output length to 1000 characters, STOUT V2.0 can now generate longer, more complex IUPAC names. This expansion is crucial for accurately naming larger molecular structures. Furthermore, we leveraged the latest TPU VMs to train the models, significantly reducing training time for these complex models with extensive datasets. These improvements in training hardware accelerate the development process and enable one to use more data and complex model architectures to train and test.

This study marks a significant step towards more accessible IUPAC name generation, yet it also opens avenues for further exploration. As large language models (LLMs) continue to evolve, future work could delve deeper into the nuances of the model's performance. Future work will focus on refining our understanding of the model's capabilities and limitations. Key areas for investigation include: identifying the simplest misnamed structures, correlating structural complexity with naming accuracy, determining accuracy thresholds for molecular complexity, assessing the impact of stereochemistry, and quantifying the contribution of edge cases. This analysis will aim to establish clear trust levels for the model's performance across various molecular categories. As large language models evolve, this research could serve as a valuable case study in applying AI to specialized chemical tasks, potentially leading to more robust tools for automated nomenclature.

Our neural network-based approach has limitations compared to rule-based systems, which can be updated by modifying the ruleset. In contrast, our model requires retraining when significant errors are found in the data, the upstream Lexichem software is updated, or when IUPAC nomenclature rules change, including revisions to the Preferred IUPAC Name (PIN) guidelines. The need for periodic retraining is a key drawback, especially in fields where nomenclature standards are frequently updated or strict adherence to the latest IUPAC guidelines is essential.

Our web application further enhances the accessibility and usability of STOUT V2, which is now accessible to users with limited programming knowledge. The application includes integrated visualisation features that enable trained chemists to identify and promptly correct minor errors in generated IUPAC names. This feature not only improves the accuracy of the output but also serves as a valuable educational tool for understanding the nuances of IUPAC nomenclature.

By making the model checkpoints, weights, and fully documented source code available as open-source resources, we aim to promote unrestricted use and encourage further development within the field. This will allow researchers in this field to build upon our work and adapt it to their specific needs.

## Data Availability

No datasets were generated or analysed during the current study.

## Tokenisation

SMILES tokenisation: The same tokenisation strategy for SMILES strings as outlined in our previous work [19], utilising the Keras [46] tokeniser for generating the token sequences. The SMILES strings were split into meaningful tokens according to the following set of rules:

- Each heavy atom (e.g., "C", "Si", "Au") was treated as a separate token.
- Each open and closed bracket (i.e., "(", ")", "[", "]") was tokenised individually.
- Each bond symbol (" = ", "#") was considered as a separate token.
- The characters ".", "-", " + ", "", "/", "@", "%", and "*" were split into individual tokens.
- Each digit was tokenised separately.

After splitting the SMILES strings into tokens, a " < start > " token was added at the beginning of each sequence, and an " < end > " token was appended at the end.

IUPAC Names tokenisation: IUPAC names were split after certain characters like opening brackets ("(", "{", "["), closing brackets (")", "}", "]"), dashes "-", periods ".", and commas ",". Additionally, they were split after specific prefixes and chemical terms like "mono", "di", "tri", "tetra", "penta", "hexa", "hepta", "octa", "nona", "deca", "oxo", "methyl", "hydroxy", "benzene", "oxy", "chloro", "cyclo", "amino", "bromo", "hydro", "fluoro", "methane", "cyano", "amido", "ethene", "phospho", "amide", "butane", "carbono", "sulfane", "sulfino", "iodo", "ethane", "ethyne", "bi", "imino", "nitro", "butan", "idene", "sulfo", "carbon", "propane", "ethen", "acetaldehyde", "benzo", "oxa", "nitroso", "hydra" and "iso". This tokenisation approach aimed to break down the IUPAC name representations into meaningful words.

IUPAC Names tokenisation Characterwise: Character-wise tokenisation involved splitting each IUPAC name into tokens after each character, with spaces also treated as characters. For example, "benzene" can be tokenised by splitting into meaningful segments or characters, resulting in different counts of unique tokens. Character-wise splitting would break "benzene" into 7 tokens with 4 unique tokens.

## Experiment 1—impact of dataset size and tokenisation on model performance:

The training dataset was obtained from the PubChem database. Of the 110 million compounds downloaded using the chemfp implementation of the MaxMin algorithm, approximately 51 million were selected. The MaxMin algorithm used here was done using the default settings.

Training and Test Sets:

- From the 51 million subset, a training dataset of 50 million compounds and a testing dataset of 1 million compounds were chosen.
- Additionally, an 11 million compound subset was selected using the MaxMin algorithm, from which a training set of 10 million compounds and a test set of 1 million compounds were created.
- A final subset of 1 million compounds for training and 250,000 for testing was also selected from the 11 million subset.

### Tokenisation procedures

SMILES tokenisation followed the methods described in Appendix 1.

IUPAC Names tokenisation: The IUPAC names were split into meaningful tokens using the tokenisation rules from Appendix 1 and were also split into tokens character-wise separately, resulting in three additional training datasets.

## Abbreviations

AIN: AI-generated IUPAC names
BLEU: BilinguaL Evaluation Understudy
CDK: Chemistry Development Kit
CPU: Central Processing Unit
GRU: Gated Recurrent Units
IUPAC: International Union of Pure and Applied Chemistry
LLM: Large Language Model
NLP: Natural Language Processing
OCSR: Optical Chemical Structure Recognition
OPSIN: Open Parser for Systematic IUPAC Nomenclature
PIN: Preferred IUPAC Names
REST: REpresentational State Transfer
RNN: Recurrent Neural Networks
SDF: Structure-Data File
SMILES: Simplified Molecular Input Line Entry Specification
STOUT: Smiles TO iUpac Translator
TFRecord: TensorFlow Record
TPU: Tensor Processing Units
V: Version
VM: Virtual Machine
XML: Extensible Markup Language

## References

[1] Favre HA, Powell WH (2014). Nomenclature of organic chemistry: IUPAC recommendations and preferred names 2013. RSC Publishing. 10.1039/9781849733069.

[2] Connelly NG, Damhus T, Hartshorn RM, Hutton AT (Eds.). Nomenclature of Inorganic Chemistry: IUPAC Recommendations 2005. RSC Publishing; 2005.

[3] Panico R, Powell WH, Richer JC (1993) A guide to IUPAC nomenclature of organic compounds: recommendations 1993 (including revisions, published and hitherto unpublished, to the 1979 edition of nomenclature of organic chemistry. Wiley-Blackwell, Hoboken

[4] Tinley EH (2013) Naming organic compounds: A guide to the nomenclature used in organic chemistry. Literary Licensing, LLC.

[5] Inczédy J, Lengyel T (1998) International union of pure and applied chemistry compendium of analytical nomenclature definitive rules 1997. Institut d’Estudis Catalans: Barcelona.

[6] Werd S. Mnova 15.0.1. https://mestrelab.com/download_file/mnova-15-0-1/. Accessed 1 July 2024.

[7] Molconvert. https://docs.chemaxon.com/display/lts-lithium/molconvert.md . Accessed 1 July 2024.

[8] Convert chemical structures and chemical names. https://www.eyesopen.com/lexichem-tk. Accessed 1 July 2024.

[9] Generate IUPAC names for chemical structures. https://www.acdlabs.com/products/name/. Accessed 1 July 2024.

[10] Website available online: ChemAxon—software solutions and services for chemistry & biology. https://www.chemaxon.com.

[11] OpenEye toolkits 2023.1. OpenEye, cadence molecular sciences, Santa Fe, NM. http://www.eyesopen.com.

[12] Dargan S, Kumar M, Ayyagari MR, Kumar G (2020) A survey of deep learning and its applications: a new paradigm to machine learning. Arch Comput Method Eng 27:1071–1092. 10.1007/s11831-019-09344-w

[13] Taye MM (2023) Understanding of machine learning with deep learning: architectures, workflow. Appl Fut Dir Comput 12:91. 10.3390/computers12050091

[14] Khan W, Daud A, Khan K, Muhammad S, Haq R (2023) Exploring the frontiers of deep learning and natural language processing: a comprehensive overview of key challenges and emerging trends. Nat Lang Process J 4:100026. 10.1016/j.nlp.2023.100026

[15] Yang S, Wang Y, Chu X. (2020) A survey of deep learning techniques for neural machine translation. arXiv [cs.CL].

[16] Brown TB, Mann B, Ryder N, Subbiah M, Kaplan J, Dhariwal P, Neelakantan A, Shyam P, Sastry G, Askell A et al. (2020) Language models are few-shot learners. arXiv [cs.CL]

[17] Chang EY. (2023) Examining GPT-4’s capabilities and enhancement with socrasynth. In: proceedings of the 2023 international conference on computational science and computational intelligence (CSCI). IEEE. Pp. 7–14. 10.1109/CSCI62032.2023.00009.

[18] Schwaller P, Gaudin T, Lányi D, Bekas C, Laino T (2018) ‘Found in translation’: predicting outcomes of complex organic chemistry reactions using neural sequence-to-sequence models. Chem Sci 9:6091–6098. 10.1039/c8sc02339e 30090297 10.1039/c8sc02339e PMC6053976

[19] Rajan K, Brinkhaus HO, Agea MI, Zielesny A, Steinbeck C (2023) DECIMER. ai: an open platform for automated optical chemical structure identification, segmentation and recognition in scientific publications. Nat Commun 14:5045. 10.1038/s41467-023-40782-0 37598180 10.1038/s41467-023-40782-0 PMC10439916

[20] Blanco-González A, Cabezón A, Seco-González A, Conde-Torres D, Antelo-Riveiro P, Piñeiro Á, Garcia-Fandino R (2023) The role of AI in drug discovery: challenges, opportunities, and strategies. Pharmaceuticals. 10.3390/ph16060891 37375838 10.3390/ph16060891 PMC10302890

[21] Ertl P, Lewis R, Martin E, Polyakov V. (2017) In Silico generation of novel, drug-like chemical matter using the LSTM neural network. arXiv [cs.LG].

[22] Olivecrona M, Blaschke T, Engkvist O, Chen H (2017) Molecular de-novo design through deep reinforcement learning. J Cheminform 9:48. 10.1186/s13321-017-0235-x 29086083 10.1186/s13321-017-0235-x PMC5583141

[23] Ivanenkov YA, Polykovskiy D, Bezrukov D, Zagribelnyy B, Aladinskiy V, Kamya P, Aliper A, Ren F, Zhavoronkov A (2023) Chemistry42: an ai-driven platform for molecular design and optimization. J Chem Inf Model 63:695–701. 10.1021/acs.jcim.2c01191 36728505 10.1021/acs.jcim.2c01191 PMC9930109

[24] Baum ZJ, Yu X, Ayala PY, Zhao Y, Watkins SP, Zhou Q (2021) Artificial intelligence in chemistry: current trends and future directions. J Chem Inf Model 61:3197–3212. 10.1021/acs.jcim.1c00619 34264069 10.1021/acs.jcim.1c00619

[25] Handsel J, Matthews B, Knight NJ, Coles SJ (2021) Translating the InChI: adapting neural machine translation to predict IUPAC names from a chemical identifier. J Cheminform 13:79. 10.1186/s13321-021-00535-x 34620215 10.1186/s13321-021-00535-x PMC8496104

[26] Rajan K, Zielesny A, Steinbeck C. (2021) STOUT: SMILES to IUPAC names using neural machine translation. J Cheminform 13(1):34. 10.1186/s13321-021-00512-4. 33906675 10.1186/s13321-021-00512-4 PMC8077691

[27] Krasnov L, Khokhlov I, Fedorov MV, Sosnin S (2021) Transformer-based artificial neural networks for the conversion between chemical notations. Sci Rep 11:14798. 10.1038/s41598-021-94082-y 34285269 10.1038/s41598-021-94082-y PMC8292511

[28] Lowe DM, Corbett PT, Murray-Rust P, Glen RC (2011) Chemical name to structure: OPSIN, an open source solution. J Chem Inf Model 51:739–753. 10.1021/ci100384d 21384929 10.1021/ci100384d

[29] Kim S, Chen J, Cheng T, Gindulyte A, He J, He S, Li Q, Shoemaker BA, Thiessen PA, Yu B et al (2023) PubChem 2023 update. Nucl Acid Res 51:D1373–D1380. 10.1093/nar/gkac956 10.1093/nar/gkac956 PMC9825602 36305812

[30] ChemAxon. Molconvert: part of Marvin Suite 20.15: Cheminformatics toolkit for structure file conversion and rendering [Software]. Available online: https://chemaxon.com. Accessed on 14 Oct 2024.

[31] Tanimoto TT (1958) An elementary mathematical theory of classification and prediction. International Business Machines Corporation, New York

[32] Molecular Modeling Software. http://www.eyesopen.com. Accessed 5 August 2024.

[33] Dalke A (2019) The Chemfp Project. J Cheminform 11:76. 10.1186/s13321-019-0398-8 33430977 10.1186/s13321-019-0398-8 PMC6896769

[34] Ashton M, Barnard J, Casset F, Charlton M, Downs G, Gorse D, Holliday J, Lahana R, Willett P (2002) Identification of diverse database subsets using property-based and fragment-based molecular descriptions. Quant Struct Act Relatsh 21:598–604. 10.1002/qsar.200290002

[35] Vaswani A, Shazeer N, Parmar N, Uszkoreit J, Jones L, Gomez AN, Kaiser L, Polosukhin I. (2017) Attention is all you need. arXiv [cs.CL].

[36] Yu T, Zhu H. (2020) Hyper-parameter optimization: a review of algorithms and applications. arXiv [cs.LG].

[37] Vue.js. https://vuejs.org. Accessed 14 Oct 2024.

[38] FastAPI. https://fastapi.tiangolo.com. Accessed 14 Oct 2024.

[39] Rajan K, Zielesny A, Steinbeck C (2020) DECIMER: towards deep learning for chemical image recognition. J Cheminform 12:65. 10.1186/s13321-020-00469-w 33372621 10.1186/s13321-020-00469-w PMC7590713

[40] Papineni K, Roukos S, Ward T, Zhu WJ. (2002) Bleu: A method for automatic evaluation of machine translation. In: proceedings of the proceedings of the 40th annual meeting of the association for computational linguistics. Pp. 311–318. 10.3115/1073083.1073135.

[41] Quinonero-Candela J, Sugiyama M, Schwaighofer A, Lawrence ND (2022) Dataset shift in machine learning. MIT Press, Cambridge

[42] Pan SJ, Yang Q (2010) A survey on transfer learning. IEEE Trans Knowl Data Eng 22:1345–1359. 10.1109/TKDE.2009.191

[43] Willighagen EL, Mayfield JW, Alvarsson J, Berg A, Carlsson L, Jeliazkova N, Kuhn S, Pluskal T, Rojas-Chertó M, Spjuth O et al (2017) The chemistry development kit (CDK) v20.: atom typing, depiction, molecular formulas, and substructure searching. J Cheminform. 10.1186/s13321-017-0220-4 29086040 10.1186/s13321-017-0220-4 PMC5461230

[44] Steinbeck C, Han Y, Kuhn S, Horlacher O, Luttmann E, Willighagen E (2003) The chemistry development kit (CDK): an open-source java library for chemo- and bioinformatics. J Chem Inf Comput Sci 43:493–500. 10.1021/ci025584y 12653513 10.1021/ci025584y PMC4901983

[45] Karulin B, Kozhevnikov M (2011) Ketcher: web-based chemical structure editor. J Cheminform 3:1–1. 10.1186/1758-2946-3-S1-P321214931

[46] Chollet, F., et al. Keras. https://keras.io.

